# Effects of graded concentrations of supplemental selenium on selenium concentrations in tissues and prediction equations for estimating dietary selenium intake in pigs

**DOI:** 10.7717/peerj.5791

**Published:** 2018-10-19

**Authors:** Ah Reum Son, Jin-young Jeong, Kyu Ree Park, Minseok Kim, Sung Dae Lee, Ji-Hyock Yoo, Yoon-Jung Do, Kondreddy Eswar Reddy, Hyun-Jeong Lee

**Affiliations:** 1 Department of Animal Science and Technology, Konkuk University, Seoul, Republic of Korea; 2 Animal Nutritional Physiology Team, National Institute of Animal Science, Rural Development Administration, Wanju-gun, Republic of Korea; 3 Department of Agro-food Safety, National Institute of Agricultural Science, Rural Development Administration, Wanju-gun, Republic of Korea; 4 Division of Animal Diseases & Health, National Institute of Animal Science, Rural Development Administration, Wanju-gun, Republic of Korea; 5 Current affiliation: Department of Animal Science, College of Agriculture and Life Sciences, Chonnam National University, Gwangju, Republic of Korea

**Keywords:** Selenium, Swine, Accumulation, Organ

## Abstract

The experiment was conducted to determine the effects of graded dietary selenium (Se) on organ weight and Se concentrations in tissues and to develop equations for estimating dietary Se intake in pigs. Sixteen barrows (initial body weight = 30.0 ± 2.6) were allotted to four dietary treatments including graded Se supplementations with 0, 1, 5, and 50 mg/kg of diet. The experimental diets fed to the pigs for 30 d, and then the pigs were euthanized, and the organs, muscle, and urine samples were collected. The hair and blood samples of pigs were collected on d 15 and 30. Equations were developed for predicting daily Se intake using the Se concentration in plasma, hair, liver, kidneys, muscle, or urine. For graded dietary Se concentrations, linear and quadratic effects on the final body weight, weight and relative weight of liver and kidneys were not observed. The Se concentration in plasma, hair, liver, kidneys, muscle, and urine were linearly and quadratically increased as dietary Se concentration increased (*P* < 0.001). The dietary Se concentration was positively correlated with the Se concentrations in the plasma, organs, muscle, and urine (*r* > 0.81, *P* < 0.001). The equations for estimating dietary Se intake using the Se concentration in the plasma, hair, or organ as an independent variable were significant (*P* < 0.05). In conclusion, the dietary Se concentration was well reflected in the Se concentration in the plasma, hair, liver, kidneys, and urine. The Se concentration in the plasma, hair, liver, and kidneys can be used as an independent variable for estimating the Se intake.

## Introduction

Selenium (Se) is one of essential trace minerals, and it plays important biochemical roles as a component of selenoproteins to prevent oxidative damages in the body (Barbosa et al., 2017). Furthermore, as part of selenoproteins, the Se modulates physiological processes related with the immune system and thyroid metabolism for pigs (Mahan, 2001; Dalgaard et al., 2018). The Se deficiency in pigs induces sudden death, reduced reproduction and milk production, and impaired immune response (National Research Council, 2012). Positive effects of supplemental dietary Se on meat quality and antioxidative status for pigs have been reported (Calvo et al., 2017; Schwarz et al., 2017). However, excessive consumption of Se occurs toxicity in animals. For the pigs, generally 5 to 25 ppm of Se in diets causes a chronic selenosis. Major symptoms of selenosis are reduced growth rate and feed intake, hair loss, hoof lesion, vomiting, and incoordination (Mahan, 2001; National Research Council, 2005).

For Se, safety margin between adequate amount and overconsumption is narrow in humans and other mammals, so it has become more important to estimate the Se intake (Reis et al., 2017; Ullah et al., 2018). In addition, deficient or excessive Se intake depends on regions because the Se concentration in vegetables can be largely affected by Se concentration in soil (Reis et al., 2017). Natural and anthropogenic sources of Se including bedrocks, volcanic activity, Se-fertilizers, and coal burning influence the Se concentration in soil (Winkel et al., 2015), and this subsequently can affect the Se concentration in plants and animal body. Moreover, livestock products have relatively high Se concentration compared with fruits and cereals (Choi et al., 2009; Ullah et al., 2018). It has been reported that Se concentration in organs and tissues of pigs increased as dietary Se concentrations increased (Goehring et al., 1984; Kim & Mahan, 2001a; Kim & Mahan, 2001b; Mateo et al., 2007). In EU and USA, supplementation of Se in complete feeds for swine is regulated at a level not to exceed 0.2 and 0.3 mg/kg, respectively. Regulatory limits for the Se in swine diets can be determined based on accumulation rate of dietary Se to edible portion of pigs. However, studies on relationship between dietary Se intake and concentration in tissues of pigs are limited.

Therefore, the objectives of this experiment were (1) to determine the effects of graded supplemental Se concentrations on body and organ weights and the Se concentrations in the plasma, hair, soft tissues (liver, kidneys, and muscle), and urine; and (2) to generate equations for estimating daily Se intake using the Se concentrations in the plasma, hair, soft tissues, or urine as an independent variable for pigs.

## Materials and Methods

The protocols used for the animal experimental procedures were reviewed and approved by the Institutional Animal Care and Use Committee of the National Institute of Animal Science, South Korea No. 2015-147 of 29 May 2015. The experimental procedure of current study was performed as described in our previous study (Reddy et al., 2017).

### Animals, diets, and experimental design

A total of 16 barrows with a mean initial body weight (BW) 30 kg (standard deviation = 2.6) was randomly assigned to four dietary treatments. A control diet without dietary Se supplementation was prepared mainly based on corn and soybean meal (Table 1). The control diet was formulated to meet or exceed the nutrient requirements estimates (National Research Council, 2012). All pigs were fed the control diet during the experimental period. The Se as a sodium selenite put into a soft capsule, and then the encapsulated Se was supplemented to additional three dietary treatment groups along with each meal (1, 5, or 50 mg/kg of diet). The encapsulated Se was mixed with each meal, and an experimenter checked and confirmed that the pigs completely consumed their feed allowance and capsule at each feeding time. An experimental period lasted for 30 d, and the daily feed allowance of each pigs was 1 kg and 2 kg for first and last 15 d, respectively. The pigs were fed two equal meals daily, and freely accessed to water.

**Table 1:** Ingredients and chemical compositions of control diet (as-fed basis).

Item	Control diet
Ingredients (%)	
Ground corn	58.56
Soybean meal (46% crude protein )	14.00
Extruded soybean meal	12.00
Whey powder (12% crude protein)	7.00
Fish meal	3.45
Soybean oil	1.60
_L_-Lysine⋅hydrochloride (78%)	0.43
_DL_-Methionine (99%)	0.14
_L_-Threonine (99%)	0.12
Monodicalcium phosphate	1.08
Ground limestone	0.60
Choline chloride (50%)	0.20
Sodium chloride	0.32
Vitamin-trace mineral premix^a^	0.50
Calculated nutrients (%)	
Metabolizable energy (kcal/kg)	3,444
Crude fiber	2.29
Crude protein	20.78
Lysine	1.47
Methionine	0.49
Calcium	0.75
Phosphorus	0.45
Selenium^b^ (mg/kg)	0.13

**Notes.**

a Provided the following quantities per kg of complete diet: vitamin A 11,000 IU; vitamin D_3_ 1,500 IU; vitamin E 44.1 IU; vitamin K_3_ 4.0 mg; vitamin B_1_ 1.4 mg; vitamin B_2_ 5.22 mg; vitamin B_5_ 20.0 mg; vitamin B_12_ 0.01 mg; Niacin 26.0 mg; Pantothenic acid 14 mg; Folic acid 0.8 mg; Biotin 44 μg; Fe 100.0 mg as iron sulfate; Cu 16.50 mg as copper sulfate; Zn 90.0 mg as zinc sulfate; Mn 35.0 mg as manganese sulfate; I 0.30 mg as calcium iodate.

b Calculated value from National Research Council (2012) and Prabhu (2015).

### Sampling and processing

On d 0 and 30, all pigs were individually weighed, and blood and hair were collected from each pig. The blood was collected from jugular vein, and then samples were taken into metal free heparinized collection tubes (BD Vacutainer^®^, Cat. No. 366480, Franklin Lakes, NJ, USA), and immediately centrifuged at 4 °C for 15 min at 2,500 rpm. The plasma samples were stored at −20 °C for until analysis. At the end of experiment, all pigs were euthanized by an anesthetic overdose with the combination of barbiturates and pentobarbital. After slaughter, the liver, both kidneys, and thigh muscle samples were collected, and then immediately frozen by using the liquid nitrogen, and stored at −80 °C. From the urinary bladder, the urine samples were collected by using syringe, and were stored at −20 °C for until analysis.

### Sample digestion procedure and elemental analysis

The liver, kidneys, and muscle samples were dried at −105 °C until constant weight is reached (Linden, Olsson & Oskarsson, 1999), and then finely ground before analysis. For the hair samples, a preconditioning process was performed according to a previous study (Chaturvedi et al., 2014). The amounts of samples used for each analysis were: 1 mL of plasma, 0.1 g of hair, 0.2 g of dried liver, kidneys, or muscle, and 1.5 mL of urine. Each sample was analyzed for the Se concentration in triplicate. Each digestion procedure included three blank samples.

The procedure of digestion and analysis was based on a previous study (Ashoka et al., 2009). A 2.5 mL of concentrated HNO_3_ and 0.5 mL of concentrated HCl were added to the samples placed in a glass tube with cap, and the tubes kept in a water bath at 85 °C. Three hours later, the samples were cooled at room temperature. After cooling, the samples were diluted to 50 mL using a 2% HNO_3_ solution (Cubadda, Raggi & Coni, 2006). The digested samples were analyzed for the Se concentration using an ICP-MS (Agilent 7500, Santa Clara, CA, USA).

### Statistical analysis

The MIXED procedure of SAS (SAS Institue Inc., Cary, NC, USA) were used to analyze experimental data. The model included the dietary treatment as an independent variable. Linear and quadratic effects of dietary Se concentrations were tested using the orthogonal polynomial contrast. To generate the contrast coefficients for unequally spaced dietary Se concentrations, the IML procedure of SAS was used. The CORR procedure of SAS was used to determine the correlation between dietary Se concentration and Se concentrations of plasma, hair, soft tissues (liver, kidneys, and muscle), and urine. The REG and NLIN procedures of SAS were used to develop linear and quadratic equations for predicting daily Se intake of the pigs. The experimental unit was the pig, and level of statistical significance (alpha level) was set at 0.05.

## Results

The final BW of pigs was not affected by dietary Se concentration (Table 2). The linear and quadratic effects on the weight and relative weight of liver and kidneys with increasing dietary Se concentration were not observed. The Se concentrations in plasma, hair, liver, kidneys, muscle, and urine were linearly and quadratically increased with increasing dietary Se concentration (*P* < 0.001; Table 3).

**Table 2:** Final body weight (BW) and weight of liver and kidneys for pigs fed the diets containing graded concentrations of supplemental selenium (Se).^a^

Item	Supplemental Se, mg/kg of diet					*P*-value	
	0	1	5	50	SEM^b^	Linear	Quadratic
Initial BW, kg	29.0	28.8	29.4	29.9	–	–	–
Final BW, kg	52.0	49.7	52.3	52.1	2.0	0.724	0.698
Weight, g							
Liver	1,672	1,658	1,870	1,772	89	0.599	0.100
Kidney	135	141	160	160	9	0.141	0.082
Relative weight to BW, %							
Liver	3.24	3.34	3.57	3.41	0.16	0.776	0.165
Kidney	0.26	0.28	0.31	0.31	0.02	0.186	0.142

**Notes.**

a Each least squares mean represents four observations.

b SEM, standard error of the means.

**Table 3:** Selenium (Se) concentrations in plasma, hair, liver, kidneys, muscle, and urine for pigs fed the diets containing graded concentrations of supplemental Se.^a^

Item	Supplemental Se, mg/kg of diet					*P*-value	
	0	1	5	50	SEM ^b^	Linear	Quadratic
Plasma, mg/L							
d 15	0.25	0.32	1.96	3.52	0.16	<0.001	<0.001
d 30	0.18	0.35	2.10	4.25	0.19	<0.001	<0.001
Hair, mg/kg							
d 15	0.42	0.45	1.06	2.42	0.08	<0.001	<0.001
d 30	0.41	0.46	1.47	5.19	0.05	<0.001	<0.001
Liver, mg/kg	9.62	16.2	41.9	83.9	2.22	<0.001	<0.001
Kidneys, mg/kg	18.5	37.3	62.8	114.9	2.4	<0.001	<0.001
Muscle, mg/kg	2.62	3.52	4.57	5.78	0.15	<0.001	<0.001
Urine, mg/L	0.06	0.19	1.01	1.47	0.08	<0.001	<0.001

**Notes.**

a Each least squares mean represents four observations.

b SEM, standard error of the means.

**Figure 1:**
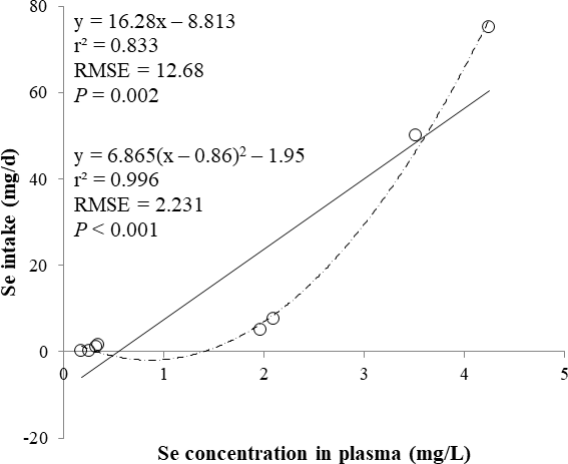
Linear and quadratic regression equations for estimating daily selenium (Se) intake (mg/d) based on the Se concentration in plasma (mg/L) on d 15 and 30. Each data point represents least squares mean of four observations. *r*^2^, coefficient of determination; RMSE, root mean square of error.

**Figure 2:**
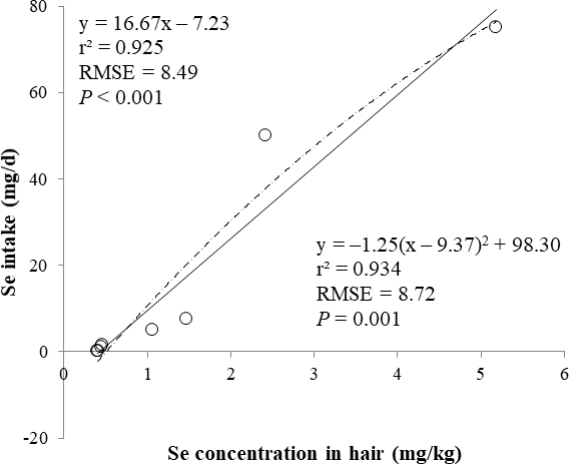
Linear and quadratic regression equations for estimating daily selenium (Se) intake (mg/d) based on the Se concentration in hair (mg/kg) on d 15 and 30. Each data point represents least squares mean of four observations. *r*^2^, coefficient of determination; RMSE, root mean square of error.

**Figure 3:**
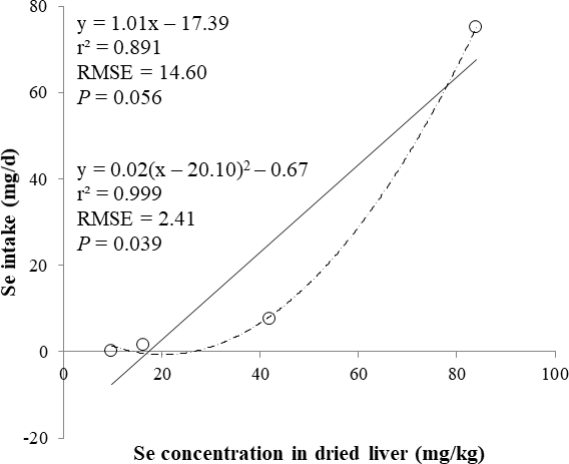
Linear and quadratic regression equations for estimating daily selenium (Se) intake (mg/d) based on Se concentration in dried liver (mg/kg). Each data point represents least squares mean of four observations. *r*^2^, coefficient of determination; RMSE, root mean square of error.

**Figure 4:**
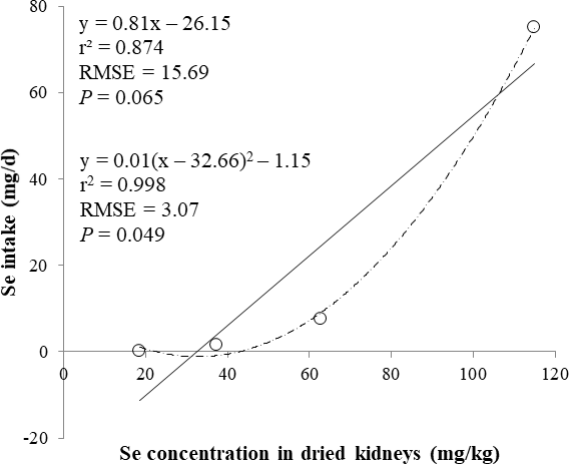
Linear regression equation for estimating daily selenium (Se) intake (mg/d) based on the Se concentration in dried kidneys (mg/kg). Each data point represents least squares mean of four observations. *r*^2^, coefficient of determination; RMSE, root mean square of error.

**Figure 5:**
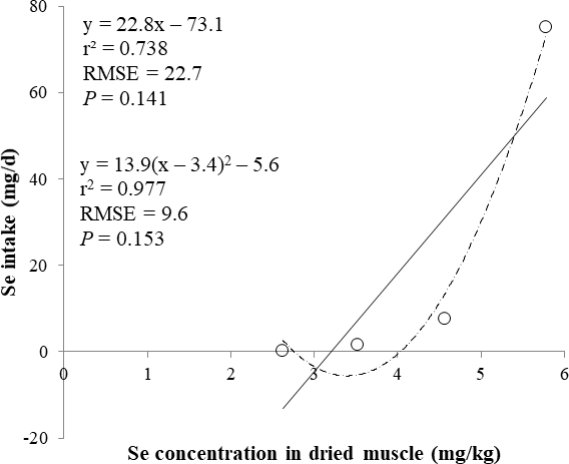
Linear and quadratic regression equations for estimating daily selenium (Se) intake (mg/d) based on the Se concentration in dried muscle (mg/kg). Each data point represents least squares mean of four observations. *r*^2^, coefficient of determination; RMSE, root mean square of error.

**Figure 6:**
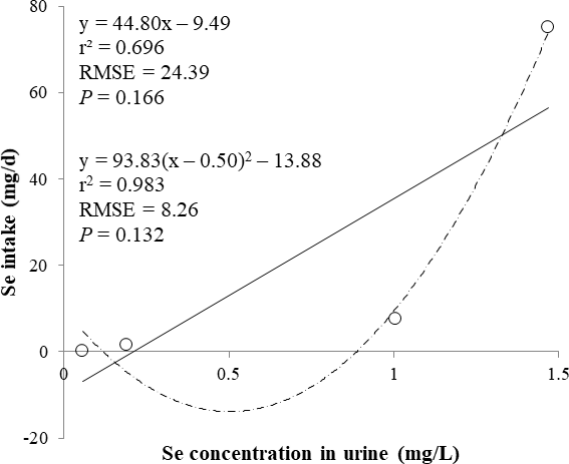
Linear and quadratic regression equations for estimating daily selenium (Se) intake (mg/d) based on the Se concentration in urine (mg/L). Each data point represents least squares mean of four observations. *r*^2^, coefficient of determination; RMSE, root mean square of error.

The correlations between the dietary Se and final BW or relative weight to BW of organ were not observed (Table 4). There were positive correlations between the dietary Se concentration and the Se concentrations in the plasma, hair liver, kidneys, muscle, and urine (*r* > 0.81, *P* < 0.001). Using the Se concentration in plasma, hair, liver, kidneys, muscle, or urine as an independent variable, the linear and quadratic equations for estimating daily Se intake were developed. The linear and quadratic equations using the Se concentration in plasma or hair were significant (*P* < 0.01; Figs. 1 and 2). The quadratic equations based on the Se concentration in the dried liver or kidneys had significance (*P* < 0.05; Figs. 3 and 4) and high *r*^2^ (0.998). There was no significance for the prediction equations developed based on the Se concentration in the dried muscle or urine (Figs. 5 and 6).

**Table 4:** Correlation coefficients (*r*) between supplemental selenium (Se) concentration and final body weight (BW), liver and kidneys weights relative to BW, and Se concentration in blood, hair, liver, kidneys, muscle, and urine.

Item	Supplemental Se concentrations	
	*r*	*P*-value
Final BW (kg)	0.100	0.713
Liver weight relative to BW	0.077	0.777
Kidneys weight relative to BW	0.343	0.194
Se concentration		
Plasma (mg/L)		
d 15	0.884	<0.001
d 30	0.909	<0.001
Hair (mg/kg)		
d 15	0.960	<0.001
d 30	0.990	<0.001
Liver (mg/kg)	0.936	<0.001
Kidneys (mg/kg)	0.929	<0.001
Muscle (mg/kg)	0.840	<0.001
Urine (mg/L)	0.810	<0.001

## Discussion

The dietary Se concentration did not affect the final BW of pigs in the present study. This result agreed with previous studies (Mahan, Cline & Richert, 1999; Mateo et al., 2007). In contrast, it was reported that the dietary Se cause decreased growth performance in the pigs (Goehring et al., 1984; Mahan & Moxon, 1984; Kim & Mahan, 2001a; Kim & Mahan, 2001b). The influence of dietary Se can be affected by source of Se, age, physical status, feeding duration, and dietary Se concentration (National Research Council, 2005). In previous studies reported decreased growth performance for pigs fed diets containing excessive Se, experimental period lasted 5 to 12 wk, and the pigs had ad libitum access to the experimental diets (Goehring et al., 1984; Mahan & Moxon, 1984; Kim & Mahan, 2001a; Kim & Mahan, 2001b). However, in the current study, the daily feed allowance of pigs was restricted at 1 or 2 kg during the 30 d of experimental period. In addition, some previous studies used weanling pigs (Goehring et al., 1984; Mahan & Moxon, 1984) which can be more sensitive to Se compared with older pigs. For these reasons, there were differences in effects of dietary Se on the growth performance between the studies.

The Se concentrations in the plasma, hair, soft tissues, and urine increased as the dietary Se level increased in the present study. The increased Se concentration in the plasma with supplementary Se was consistent with results of previous studies fed either sodium selenite or organic Se to the pigs (Kim & Mahan, 2001a; Zhan et al., 2007; Zhou et al., 2009). Normal Se concentration in serum of pig ranges from 0.08 to 0.15 mg/L (Mahan, 2001). However, the Se concentration in plasma for pigs fed the control diet in the present study was slightly greater than the previous studies (0.22 vs. 0.06 to 0.12 mg/L; Kim & Mahan, 2001a; Zhan et al., 2007; Zhou et al., 2009).

The normal Se concentration in the tissues of pigs is kidney > liver > glandular tissue > muscle in declining order of abundance (Mahan, 2001). In the current study, the kidney and liver had also higher Se concentration regardless of dietary Se level. This result indicated that ingested Se was mainly retained the kidney and liver for the pigs. In the previous studies (Mahan & Moxon, 1984; Kim & Mahan, 2001a; Kim & Mahan, 2001b; Mahan et al., 2005), relatively high Se concentration in the kidney and liver compared with other tissues was also observed in pigs fed diets with or without Se supplementation. In this study, the hair collected on d 30 had higher Se concentration rather than that collected on d 15. It was also reported that the Se concentration in the hair increased as feeding period of dietary Se was extended from 4 to 12 wk for both inorganic and organic Se supplementation (Kim & Mahan, 2001b). Although the feeding period of dietary Se was shorter compared with the literature, the result may confirm that the Se concentration in the hair is affected by the feeding duration of Se-containing diet.

In the current study, the prediction equations for estimating daily Se intake based on the Se concentration in the plasma, hair, soft tissues, or urine were developed. A high correlation of dietary Se to various tissues (loin, liver, heart, and hair) Se concentration was reported (Mahan et al., 2005). The coefficient of determination (*r*^2^) of prediction equations developed in present study was greater than 0.83 for the plasma, hair, liver, and kidney. This result may indicate that the Se concentration in the plasma, hair, liver, and kidney is used as an independent variable for estimating daily Se intake for the pigs. Moreover, the Se concentration in the plasma and hair can be used as an antemortem biomarker of dietary status for the pigs.

In conclusion, the dietary Se concentration was highly correlated with Se concentration in the plasma, hair, liver, kidney, muscle, and urine. The Se concentration in the plasma, hair, liver, and kidneys can be used as good indicators for estimating the Se intake. The prediction equations for estimating Se intake developed in present study may be useful to determine regulatory limits for Se concentration in swine diets.

##  Supplemental Information

10.7717/peerj.5791/supp-1 Data S1 Supplemental dataset file

## Additional Information and Declarations

### Data Availability

The following information was supplied regarding data availability: The raw data are provided in a Data S1.
